# Novel ligands of Choline Acetyltransferase designed by in silico molecular docking, hologram QSAR and lead optimization

**DOI:** 10.1038/srep31247

**Published:** 2016-08-10

**Authors:** Rajnish Kumar, Bengt Långström, Taher Darreh-Shori

**Affiliations:** 1 Center for Alzheimer Research, Karolinska Institutet; Department of Neurobiology, Care Sciences and Society; Division of Translational Alzheimer Neurobiology, NOVUM, 4^th^ Floor, 141 86 Stockholm, Sweden; 2 Department of Chemistry, Uppsala University, Uppsala, Sweden

## Abstract

Recent reports have brought back the acetylcholine synthesizing enzyme, choline acetyltransferase in the mainstream research in dementia and the cholinergic anti-inflammatory pathway. Here we report, a specific strategy for the design of novel ChAT ligands based on molecular docking, Hologram Quantitative Structure Activity Relationship (HQSAR) and lead optimization. Molecular docking was performed on a series of ChAT inhibitors to decipher the molecular fingerprint of their interaction with the active site of ChAT. Then robust statistical fragment HQSAR models were developed. A library of novel ligands was generated based on the pharmacophoric and shape similarity scoring function, and evaluated *in silico* for their molecular interactions with ChAT. Ten of the top scoring invented compounds are reported here. We confirmed the activity of α-NETA, the only commercially available ChAT inhibitor, and one of the seed compounds in our model, using a new simple colorimetric ChAT assay (IC_50_ ~ 88 nM). In contrast, α-NETA exhibited an IC_50_ of ~30 μM for the ACh-degrading cholinesterases. In conclusion, the overall results may provide useful insight for discovering novel ChAT ligands and potential positron emission tomography tracers as *in vivo* functional biomarkers of the health of central cholinergic system in neurodegenerative disorders, such as Alzheimer’s disease.

Dementia is a leading cause of death affecting more than 47.5 million people worldwide with additional 7.7 million new cases every year and expected to increase to 75.6 million by 2030^1^. Alzheimer’s disease (AD) alone accounts for nearly 60–70% cases of dementia. Other forms include vascular dementia, dementia with Lewy bodies (DLB) and frontotemporal dementia and Down syndrome (DS).

One of the common hallmarks of AD, DLB and DS disorders is an early selective degeneration of cholinergic neurons^2^,^3^,^4^. Expression of the acetylcholine (ACh)-synthesizing enzyme, Choline acetyltransferase (ChAT; choline O-acetyltransferase, EC: 2.3.1.6) defines theses neurons. This enzyme catalyzes reversibly the transfer of acetyl group from acetyl-Co A to choline for synthesis of acetylcholine (ACh). The central cholinergic system consists of four “ChAT-containing” neuronal nuclei (Ch1-Ch4) in the basal forebrain^5^. Ch1 and Ch2 innervate the hippocampal complex, Ch3 the olfactory bulb and Ch4 the rest of cerebral cortex and amygdala^5^. Ch4-neurons are located in the nucleus basalis of Meynert (NBM). Histopathological analyses show 75–98% reduced levels of ChAT in brain regions that become affected early and severely in dementia^6^,^7^, such as the regions of medial temporal lobe^8^, involved in memory consolidation. This leads to the use of cholinesterase inhibitors with the rational of prolonging the action of ACh at the downstream targets of the remaining cholinergic projections. The targets of these drugs are acetylcholinesterase (AChE) and butyrylcholinesterase (BuChE). These two enzymes are responsible for degrading ACh, released into synaptic cleft and/or into extracellular fluids. Cholinesterase inhibitors are as today the main therapeutic agents with proven clinical symptomatic effect albeit modestly. Up to date, other therapeutic strategies have failed to show clinical effect.

An unmet need in the dementia field is the lack of a biomarker addressing the health of CNS, in particular of the cholinergic neuronal projections. In this context, *in vivo* brain imaging by positron emission tomography (PET) with specific tracers are gaining important clinical applications and is already proven to be invaluable translational research tool for understanding early pathological events. In the past decade a number of radiolabeled amyloid beta (Aβ) imaging agents have been developed as derivatives of specific dyes such as Congo red and thioflavin-T. They are targeted at fibrillar deposits of Aβ peptides in the AD brain. Pittsburgh compound B (PIB) is such an example of the preparation of a^11^C-labled Aβ tracer that is frequently used in clinical diagnosis of AD^9^. There are also currently several tracers under development that are targeting pathological aggregates of tau protein, which is another key feature of the AD brain.

Nonetheless, these tracers are capable to document the chronic build-up of Aβ or tau deposits in the brain rather than the acute phase events that are affecting the function and health of CNS. Thereby the core cholinergic enzyme ChAT might be of high interest for development of an *in vivo* functional bio-imaging marker. Recent research has extended the role of cholinergic signaling from merely in neurotransmission to anti-inflammatory pathways^10^ and diverse biological processes and disorders such as cancer^11^. Research on targeting ChAT for development of new ligands is thus of interest in elucidating the role of cholinergic signaling, and thereby need to be revived.

Despite the fact that this enzyme was discovered about a century ago^12^, remarkably few ChAT ligands are known, all of which act as inhibitors of the enzyme. These include derivatives of naphthylvinylpyridine, stilbazole, alkylaminoethyl esters and 2-(α-naphthoyl) ethyltrimethylammonium iodide (α-NETA)^13^. The major limitation of the most studied class of naphthylvinylpyridines compounds is that a quaternary amine is part of the structure, which poses in most cases poor brain permeability and thus limited applicability^14^.

Advances in computational methodologies have significantly improved discovery of new leads, and their transformation into clinically useful drugs^15^. This together with the recently resolved crystal structure of human ChAT, showing histidine (HIS_324_) as the major catalytic amino acid residue at the center of catalytic tunnel^7^,^16^, have provide the opportunity to perform systematic analysis for discovery of new potential ChAT ligands.

The Quantitative Structure Activity Relationship (QSAR) studies are popular due to their manifold contribution in successful design of several drug candidates in account of saving time, money and human resources^17^. Generally speaking, *in silico* QSAR methods are based on assumption that certain molecular descriptors correlate with the variation in the biological activity of a series of structurally similar compounds^18^. The major challenge in developing QSAR models is to identify specific sets of molecular descriptors which correlate well with the biological activities, such as enzyme inhibition.

Hologram QSAR (HQSAR) is a fragment based QSAR method, in which the structure of a molecule is subdivided into various components, such as bonds, chirality, donor/acceptor etc. Combinations of these components are then used to design a hologram, as structural fingerprints correlating with the biological activity of dataset molecules and to generate statistical models for predicting the activity of novel compounds with similar scaffold^19^,^20^.

In the present study a specific strategy is reported for the design of novel ChAT ligands based on HQSAR and molecular docking analyses on a dataset of 26 ChAT inhibitors with reported IC_50._ The results provide insight into the contribution of specific structural moieties of the compounds towards their activity on ChAT, which allowed us to select sixteen most potent compounds for use as reference and seed structures to generate novel ligands based on the pharmacophoric and shape similarity scoring function. The designed analogues were analyzed for their molecular interactions with the active site amino acid residues of ChAT using molecular docking and the top scoring compounds with overall high score are reported here. Additionally, we also performed *in vitro* evaluation of α-NETA, one of the only commercially available potent ChAT ligand using our in house developed colorimetric assays of ChAT, AChE and BuChE. Overall the results may be useful for design and discovery of novel and potent ChAT ligands and development of ChAT PET tracers for early diagnosis of Alzheimer Disease and other neurodegenerative disorders.

## Results

### Development of highly predictive Hologram QSAR models

HQSAR is a fragment based QSAR method which correlates structure based molecular fingerprints with the biological activity of dataset molecules and results in statistical models for predicting the activity of novel compounds with similar scaffold^19^,^20^.

First, structures along with biological activity of dataset of 26 known potent ChAT ligands (Table 1) were extracted from literature. Then statistical HQSAR models were prepared by using various components such as atoms (A), bonds (B), connections (C), hydrogens (D), chirality (Ch), donor-acceptor (DA). This approach implemented different combinations of the components (A/B, A/B/C, A/B/C/H, A/B/C/Ch, A/B/C/H/Ch, A/C/DA, A/B/C/H/DA, A/B/H and A/B/H/DA) with fragment size of 4–7 atoms, by partial least square regression, and were cross-validated by leave-one-out (LOO) methods^21^.

**Figure 1:**
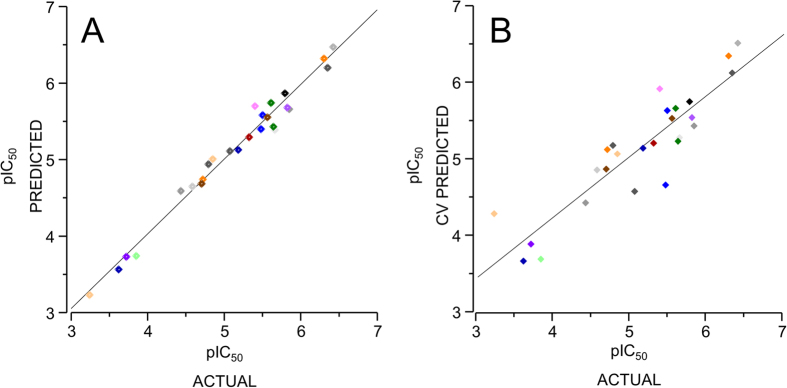
Plot of the actual pIC_50_ values versus the corresponding predicted pIC_50_ (**A**) and the cross validated (CV)-predicted pIC_50_ (**B**). The predicted pIC_50_ and CV-predicted pIC_50_value were obtained using best HQSAR model (A/B/C/Ch). The actual value corresponds to experimentally determined pIC_50_ using biological assay.

**Figure 2:**
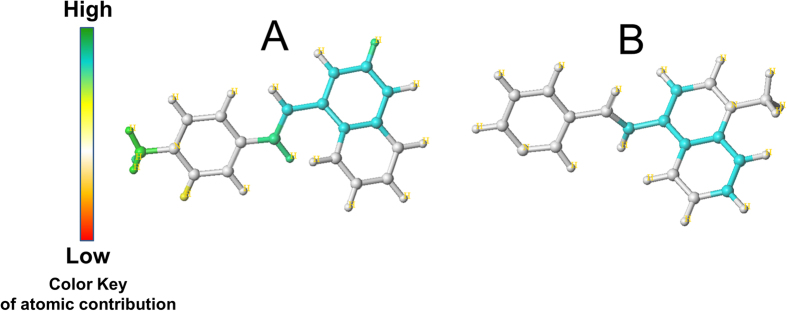
Fragment contribution map for the most potent and least potent compounds obtained using HQSAR. The N-methyl group on the pyridine ring is shown as green, which indicates a high positive contribution of this moiety for activity of the most active compound from the dataset (**A**). Removal of this group led to the loss of the activity as represented by the least active compound (**B**).

The results are summarized in Table 2.The best statistical parameters were obtained for the model no. 04, which was based on A/B/C/Ch fragments combination. This model resulted in LOO cross validated (CV) correlation coefficient (q^2^) of 0.809 and non-cross validated correlation coefficient (r^2^) of 0.977, along with the best hologram length of 151 and six components. The predictive strength of best HQSAR model (A/B/C/Ch) was then assessed by predicting the pIC_50_ value of dataset compounds. The actual pIC_50_, the predicted pIC_50_ and the CV-predicted pIC_50_values for the 26 compounds are given in Table 1. The correlations between the actual and the predicted pIC_50_ values are graphically depicted in Fig. 1. The high correlation between actual pIC_50_ and predicted pIC_50_ values indicated the good predictive ability of the developed HQSAR models. Indeed, the beauty and strength of HQSAR lies in its capability to provide information about individual atomic contribution to the biological activity using a color scale. The atomic contribution map for most active and least active compound is depicted in Fig. 2. As depicted, specific color is assigned to each atom according to its contribution towards the activity. Negative gradient contribution is assigned by red, red-orange, and orange color, while gradient of positive atomic contribution is assigned by yellow, yellow-green, and green. The atoms with neutral contribution are represented as white (see color key in Fig. 2). The N-methyl group on the pyridine ring was found essential, while the linker between the naphthyl group and the pyridine ring had high contribution for activity of the most active compound from the dataset (Fig. 2A). Removal of the N-methyl group led to loss of the activity as represented by the least active compound (Fig. 2B).

### Bio-molecular docking revealed the structural basis of the binding affinity of ChAT ligands

In order to probe the mechanism of interaction of the ChAT ligands, all the compounds from the dataset were docked into the active site of the enzyme using Surflex-Dock GeomX (SFXC) module of SYBYL-X2.1.1 suite^22^. Docking was performed utilizing the crystal structure of ChAT (PDB ID: 2FY3)^7^.The docking site was defined using coordinates of co-crystallized choline molecule. As a first step of docking, a validation of docking protocol was performed by re-docking the co-crystallized ligand in the active site of the enzyme. The RMSD between co-crystallized conformation and docked conformation has a value of 0.63 Å which indicated the suitability of docking protocol.

All of the 26 compounds in the dataset were docked into the active site of ChAT, and the docking score (Total-Score), which represent –logK_d_ are given in Table 3. The compounds with –logK_d_value larger than 6 were deemed to be the most active, represented by the compounds **1**, **13**, **18**, **20**, and **24**, among which compound **24** exhibited the highest Total-Score (−logK_d_ of 8.12).

**Figure 3:**
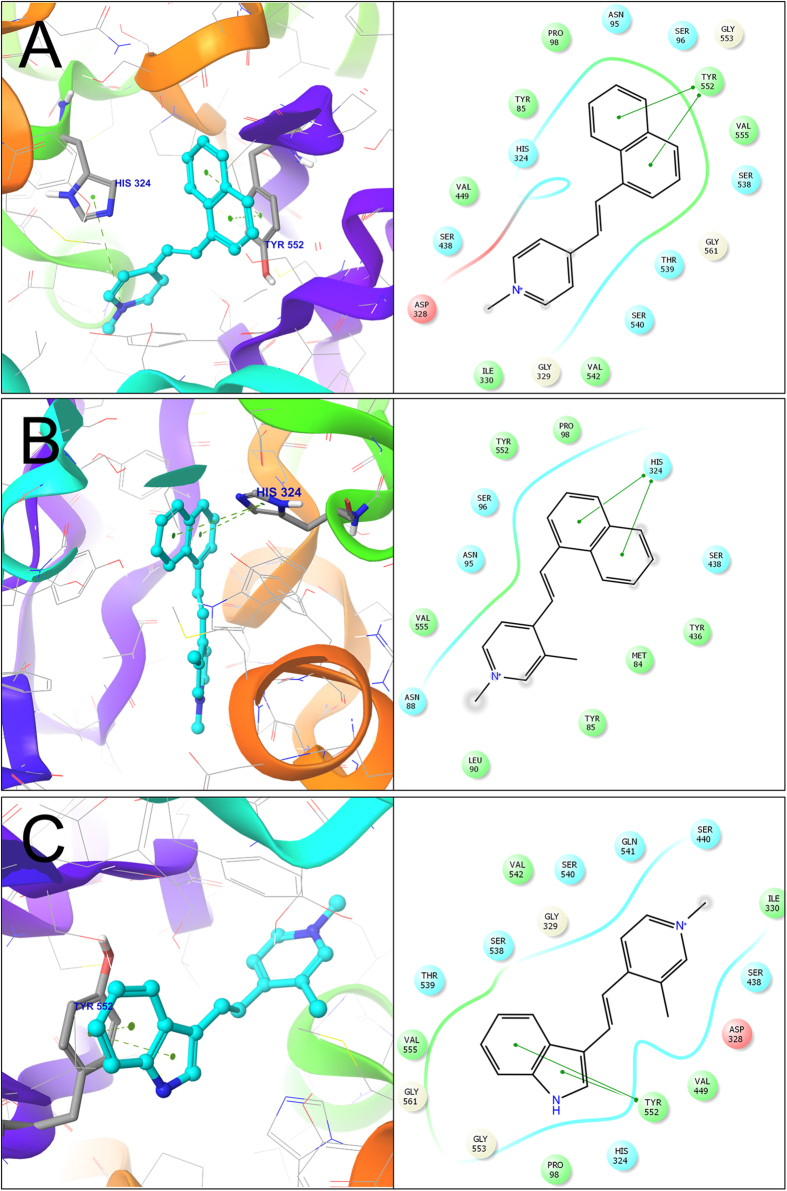
Three-dimensional docking poses of the compounds 1, 13 & 18 (**A**–**C**, respectively). The residues forming the potential interactions are given as stick model. The 2D ligand-interaction diagram illustrates the major interactions between the ligand and the active sites amino acid residues of ChAT.

The 3D docked pose and 2D ligand interaction diagram of the compounds **1**, **13**, and **18** is shown in Fig. 3(A–C). As illustrated in the Fig. 3A, the naphthyl group of compound **1**form π-π interactions with the **TYR**_**552**_ amino acid residue in the active site of ChAT. The nitrogen of the pyridine group was involved in a cation-π interaction with the **HIS**_**324**_ which is the catalytic amino acid responsible for transfer of acetyl group from A-CoA to choline. In addition, The TYR_85_, ASN_95_, SER_96_, VAL_555_, SER_538_, and GLY_561_ residues seemed to form a pocket to accommodate naphthyl group.

The top scoring pose for the compound **13** (Fig. 3B) on the other hand indicated that the naphthyl group was directly involved in π-π interactions with the main catalytic amino acid residue **HIS**_**324**,_ while the pyridine ring was extended away from the catalytic pocket and was surrounded by TYR_85_, LEU_90_ and ASN_88_ residues. The indole group of compound **18** (Fig. 3C), however like the compound **1** formed π-π interactions with **TYR**_**552**_, while **HIS**_**324**_ was one of residues that together with PRO_98_, GLY_553_, GLY_561_, VAL_555_, THR_539_ amino acid residues surrounded the indole moiety of the compound **18**. The pyridine nucleus was in vicinity of SER_438_, ILE_330_, SER_440_, and GLN_541_ residues, which might indicate that they are involved in 3D orientation of the active compound.

**Figure 4:**
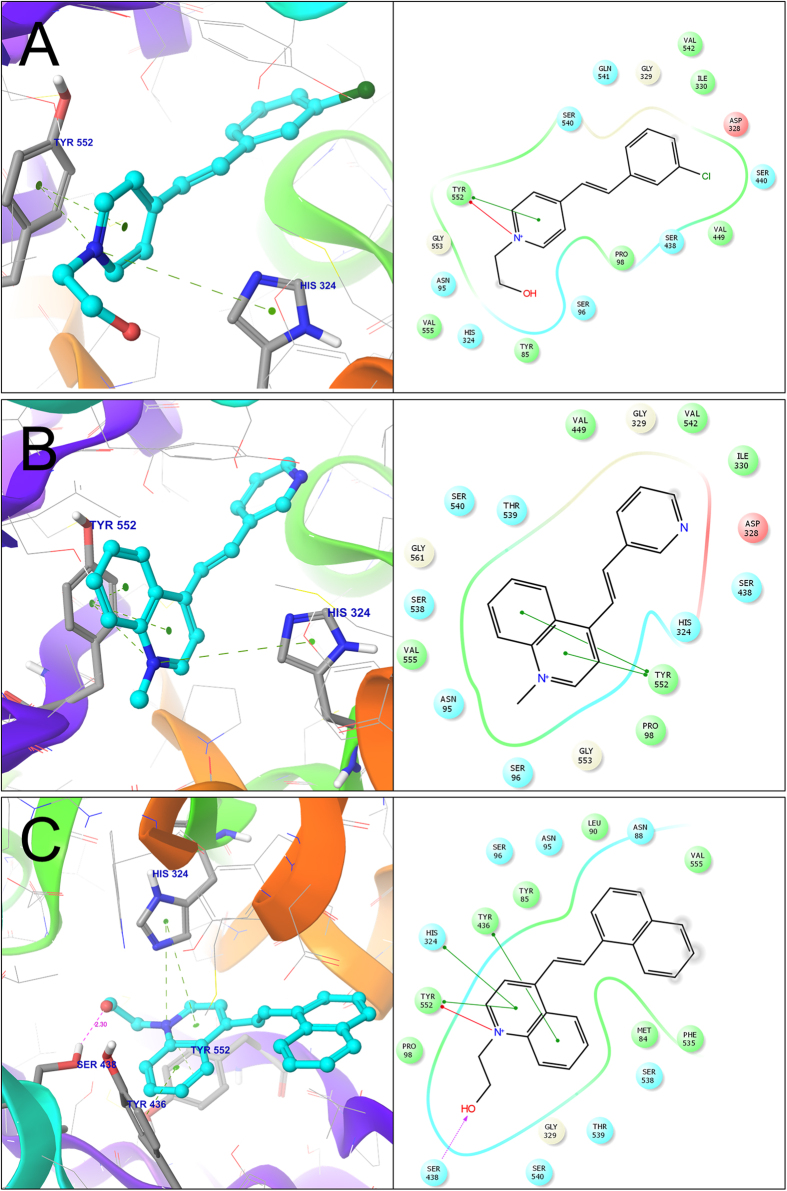
Three-dimensional docking poses of the compounds 20, 23 & 24 (**A**–**C**). The residues forming the potential interactions are given as stick model. The 2D ligand-interaction diagram illustrates the major interactions between the ligand and the active sites amino acid residues of ChAT.

The binding mode of compound **20** differed relative to the aforementioned compounds (Fig. 4A). In this case, the pyridine nucleus seemed tightly sandwiched between the**TYR**_**552**_ and **HIS**_**324**_ residues by π-π and π-cation interactions, most likely indicating their role in stabilizing the active conformation. The Phenyl-moiety of this compound was accommodated in a cavity formed by SER_440_, ASP_328_, ILE_330_, GLY_329_, and GLN_541_ residues.

In the case of compound **23** (Fig. 4B), the azanaphthyl group interacted with both the **TYR**_**552**_ (π-π interaction) and **HIS**_**324**_ residues (π-cation interaction). For compound **24**, the most active compounds with a pIC_50_ value of 6.28 (Fig. 4C), the docking pose once again includes interaction with these two amino acid residues. In this case, a 4-azanaphthyl group seemed become stabilized in 3D (Cartesian) space through formation of a π-π interaction with **HIS**_**324**_ and TYR_436_, and π-cation interactions with **HIS**_**324**_ and **TYR**_**552**_ residues. In contrast to all other active compounds, a hydroxyl group was present, which formed a hydrogen bond with SER_438_ residue at a distance of 2.30 Å. This additional interaction might be the reason behind the overall high activity of compound 24 toward ChAT.

The compound **26**, (2-(α-naphthoyl) ethyltrimethylammonium iodide) was of particular interest since this is the only ChAT ligand that currently is commercially available, and is known as (α-NETA)^13^. For α-NETA, the naphthyl group formed π-π interaction with **TYR**_**552**_, while the quaternary trimethyl ammonium moiety was closely surrounded by **HIS**_**324**_, PRO_98_, ASP_328_ residues. The naphthyl moiety was instead accommodated in a pocket consisting of ASN_95_, PRO_554_, GLY_553_, THR_539_, and SER_538_ residues.

Thus the overall pattern seemed to suggest that the **TYR**_**552**_ and **HIS**_**324**_ amino acid residues are of outmost importance for stabilization of an active conformation of ligands of ChAT.

### *In vitro* assessment of α-NETA as a potent selective ChAT inhibitor

**Figure 5:**
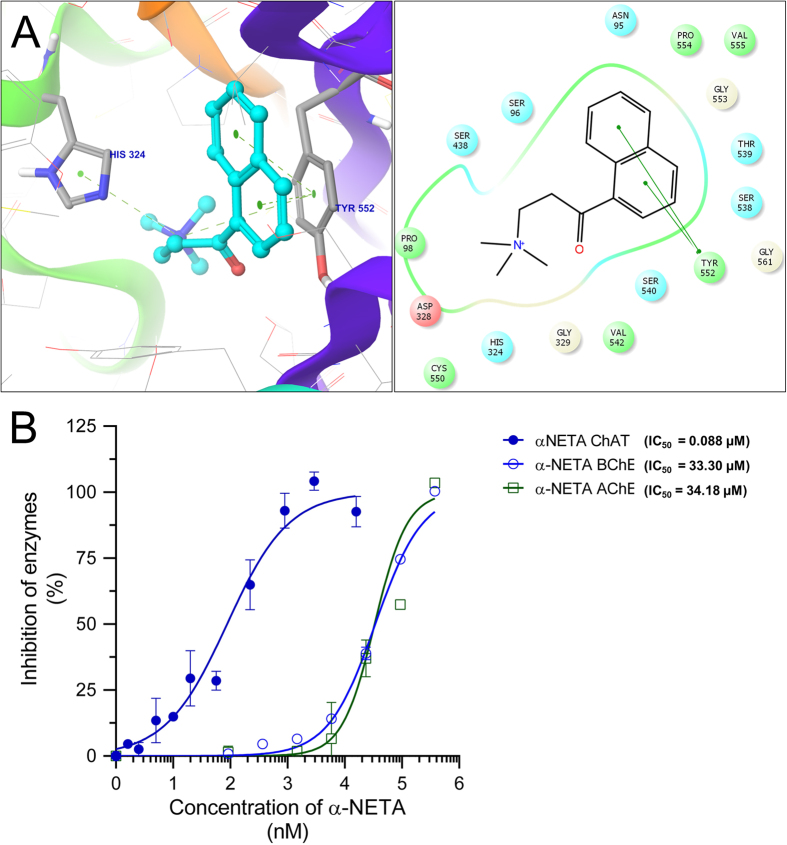
Three-dimensional docking poses of α-NETA (**A**) and its corresponding 2D ligand-interaction diagram. The 2D ligand-interaction diagram illustrates the major interactions between the ligand and the active sites amino acid residues of ChAT. (**B**) shows the *in vitro* dose-response curves for α-NETA against ChAT, acetylcholinesterase (AChE) and butyrylcholinesterase (BuChE). α-NETA was pre-incubated at the specified concentrations for 30 min with samples. The enzyme activities were measured as described in the methods section. The effect of α-NETA on the cholinergic enzymes was compared to the activity of a control sample that was preincubated with the buffer. The IC_50_ values were calculated using GraphPad Prism.

As noted, α-NETA was the only compound available commercially. This allowed us to challenge and somewhat validate our approach. Purified recombinant human ChAT protein was prepared and used to test the activity of α-NETA in a recently developed in-house colorimetric assay of ChAT. In these *in vitro* analyses, α-NETA exhibited potent inhibitory activity of ChAT, with an estimated IC_50_ of 88 nM (Fig. 5B). Since the ChAT counteracting, enzymes, AChE and BuChE apparently share affinity for acetylcholine, we deemed important to determine the selectivity of α-NETA. The determined IC_50_ values were 34.18 μM for AChE and 33.30 μM for BuChE. This indicated that α-NETA had 388 fold higher selectivity for ChAT compared to AChE, and ~378 fold relative to BuChE (Fig.5B).

### Design of novel library of ChAT ligands by Lead optimization

Next the chemical structure of the aforementioned top scoring compounds was used to generate novel analogues, using Muse^®^ Invent™ molecular design package. Briefly, the algorithm uses seed structures of the lead compounds as input, which are modified stepwise into novel analogues and rank them according to choice of scoring function^23^. For this procedure all compounds with a pIC_50_ value greater than five were selected, resulting in a set of 16 references and seed structures. This algorithm produces not only new structures with high pharmacophoric and 3D shape similarity relative to the parent structures, but also generate new compounds with moderate structural similarity to the reference compounds^24^. Through stepwise cycles this algorithm may hence generate completely new class of ligands. Here, this procedure generated a library of 996 novel potential ChAT ligands through 50 stepwise generated selection cycles.

To get insight into their interaction with ChAT’s active site amino acid residues, the generated library were subjected to molecular docking using the approach described before. The docking score for ten top-scoring compounds along with other parameters is given in the Table 4. The other parameters were those determining the BBB permeability and included hydrogen bond acceptor count, hydrogen bond donor count, rotatable bond count, total polar surface area (TPSA), and a calculated partition coefficient (ALogP).

**Figure 6:**
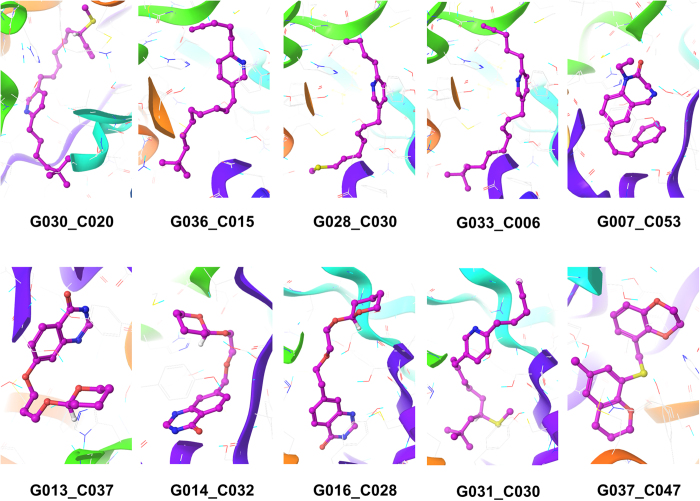
3D docked conformation of ten novel top scoring invented compounds in the active site of ChAT. The compounds are rendered as ball and stick.

The 3D docked pose of these ten top scoring compounds are illustrated in the Fig. 6.

## Discussion

Here we described generation of novel ChAT ligands from a dataset of 26 active compounds. In the course, predictive HQSAR models based on the 26 active compounds was developed, which revealed the fragments essential for the activity of these ChAT ligands. Bio-molecular docking analysis then demonstrated the most frequent amino acids residues on the active site of ChAT together with functional groups in the active compounds. In this context, the **Tyr552** and **His324** were shown to be the two amino acids forming π-π and/or π-cation interactions with certain functional moiety of the ChAT ligands.

ChAT is the enzyme responsible for synthesis of acetylcholine, one of the most important signaling substances widely used by nature in diverse physiological processes. ChAT defines cholinergic neurons which are selectively affected in AD and DLB disorders^7^, and may hence be a potential biomarker of neurodegenerative dementia disorders. Development of novel potent ChAT ligands may thereby be useful as PET tracers to study the health of CNS and neurodegeneration and/or for diagnostic purposes.

However, none of these compounds are useful as PET tracer of cholinergic network in the brain due to their instability and/or BBB impermeability. Thereby we used the insights from the HQSAR and consequential molecular docking and selected sixteen most potent ChAT ligands as seed structure and reference compounds. A virtual library of 996 new ligands was generated and the top scoring analogues are reported here. Importantly, the new ligands had high shape and pharmacophore similarity but relatively low structural resemblance to the parent compounds.

In the current analyses *Hologram QSAR* was employed. This is a specialized fragment based methods where the binding affinity of the compounds are determined on the basis of their molecular fragments^19^,^20^. The predictive power of developed models was based on LOO method, which is one of the most used methods. The best model developed contained the combination of A/B/C/Ch components, which gave highest cross validated correlation coefficient (a Q^2^ value of 0.809). Our Hologram QSAR results are in close agreement with an earlier study based on CoMFA and CoMSIA modeling^25^. For instance in agreement with our findings, their results indicated that a pyridine ring, which is one of the most common functional group among the parent compounds, had major contribution towards the affinity for ChAT. Nonetheless, *Hologram QSAR* modeling has several advantages compared to CoMFA and CoMSIA modeling. *Hologram QSAR* models in this study are not only stable and highly predictive but also avoid additional steps, such as alignment and approximation of biologically active conformation of the compound which are necessary in CoMFA and CoMSIA modeling.

In our modeling the crystal structure of a recombinant human ChAT(PDB id: 2FY3) that has quite recently reported together with co-crystallized choline as natural ligand into the active site of ChAT was used^7^. This information was crucial and allowed validation of our docking protocol by redocking of choline, resulting in optimal root mean square deviation (RMSD) between its actual conformation and docked conformation. As was noted, the biomolecular docking of the most active compounds reported in literature revealed that **HIS**_**324**_ is one of the most important amino acid residues for binding of these ligands to the active site of ChAT. Interestingly, **HIS**_**324**_ is located in the middle of catalytic tunnel of the enzyme, and is reported to be responsible for transfer of acetyl group from acetyl-CoA to choline and synthesis of acetylcholine.

The compound α-NETA was the only parent compound which was commercially available. Although it has been reported as a ChAT inhibitor, there seem to be a controversy because it was initially tested on acetyltranferase found in seminal fluids, which are considered to be carnitine acetyltransferase rather than ChAT. In our re-docking analyses, α-NETA had a Total-score (−logK_d_) of 5.3 and a reported pIC_50_ of 5.05 (an IC_50_ of ~9 μM) on purified human placental ChAT^13^.To check our model the IC_50_ of α-NETA by a recently reported high-throughput colorimetric ChAT assay, using recombinant human ChAT protein was determined. An IC_50_ of 88 nM was found, which suggest ~100 folds higher potency for α-NETA as ChAT inhibitor in our system. Furthermore, α-NETA is reported to produce 10% inhibition of AChE at 1 mM concentration^13^. In our hand, α-NETA produced a 10% inhibition of AChE at a concentration of 10 μM and BuChE at a concentration of 3 μM. However, α-NETA exhibited an IC_50_ of 48.60 μM against AChE and 33.30 μM for BuChE, once again using a high-throughput colorimetric AChE and/or BuChE assay. Nonetheless based on IC_50_ ratios, α-NETA exhibited ~388 fold and ~378 fold higher selectivity for ChAT in our *in vitro* analyses compared to AChE and BuChE, respectively. Thus our report confirms that α-NETA is a highly selective ChAT inhibitor.

In any case, α-NETA had a Total-score of 5.3, while the top ten new designed analogues reported in the current study had a Total-score of over 8 which may suggest a much higher potency than α-NETA. This warrants further development of these analogues as new classes of ChAT ligands. In addition, we included several filtering criteria such as BBB permeability, accessibility for radiolabeling and pan assay interference compounds (PAINS) filter^26^, which altogether may prove them useful as ChAT PET tracers.

In addition an updated protocol for the colorimetric ChAT assay which can easily be adopted to run on 384-wells microtiter plates, with proven suitability as high throughput *in vitro* screening tool of new ChAT ligands was applied. Nonetheless, it should be noted that the PAINS filter criteria is important since it should reduce the selection of hits that may act as unspecific multi-target inhibitors of diverse enzymes^26^.The described ChAT assay uses two axillary enzymes, namely horseradish peroxidase and choline oxidase as detection system. Thereby the PAINS filter is important to reduce possible interferences of the hits with our *in vitro* screening assay.

**Figure 7:**
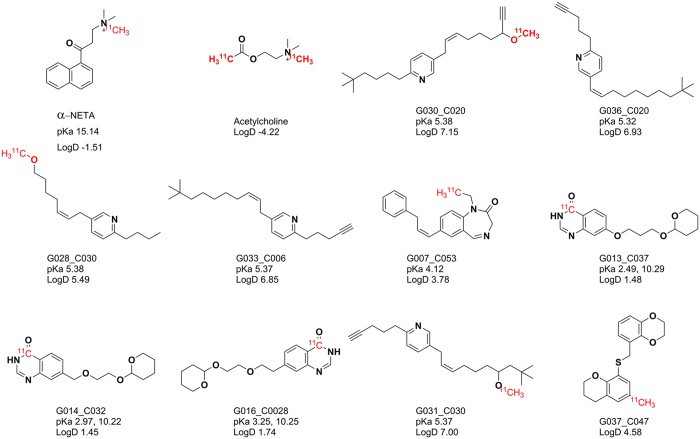
Radiolabeling feasibility of the invented ChAT ligands. The possible radiolabeling position is highlighted in red. The structures of α-NETA and Acetylcholine are also given for structural similarity comparison. Marvin was used for calculating pKa/Log D @ pH 7.4 values for the compounds, Marvin 15.4.13.0, 2015, ChemAxon (http://www.chemaxon.com).

The ease of radiolabeling is an essential requirement for developing a PET tracer. Keeping this in view, we analyzed the designed novel compounds for their radiolabeling feasibility. As shown in Fig. 7, it was observed that most of the designed compounds contain a chemical group which can be easily radiolabeled with ^11^C which is the most common radiolabeling isotope.

In conclusion, we report here a novel strategy to design novel ChAT ligands which can be developed into PET tracers with a potential to monitor the therapy and also probe the mechanism of neurodegeneration in AD and other dementia disorders.

## Methods

### Computational details

The molecular modeling studies including molecular docking and HQSAR analysis were performed using SYBYL-X 2.1.1 molecular modeling suite^22^ installed on Linux based Dell Precision T7610 workstation [Intel^®^ Xeon^®^ E5-2643 CPU @ 3.3 GHz; 16 GB RAM, 2 TB hard disk]. Muse® Invent™ molecular design workflow was utilized for ligand optimization and design of novel analogs.

### Dataset

The dataset compounds were obtained from the literature with their ChAT inhibitory potency determined using the same protocol to obtain a good QSAR model. The given molar inhibitory concentration was converted to negative logarithmic scale (PIC_50_) of the compounds and was used as a dependent variable for HQSAR. In addition, we also included α-NETA^13^ in the dataset molecule as it is the only commercially available, potent ChAT inhibitor. The 2D structure of the molecules was drawn, converted to 3D and followed by energy minimization with gradient descent method. The energy was minimized using Confort module of SYBYL X 2.1.1. The final 3D global minima conformation of the molecules was used for molecular docking studies as well for development of HQSAR models.

### Molecular docking

Docking was performed utilizing the crystal structure of ChAT (PDB ID: 2FY3)^7^ to get insight into the binding mode of dataset compounds in the active site of ChAT and performed with the Surflex-Dock module interfaced in SYBYL-X 2.1.1 which is a fully automatic flexible molecular docking algorithm with a combination of empirical scoring function and a surface based molecular similarity based search engine^27^. Prior to docking, the 3D structure of receptor was prepared as it is very common that the crystallographic data can have several problems in the structures which are needed to be fixed before molecular simulation^28^. The process of protein preparation includes addition of hydrogens, repairing side chain, treating termini, fixing atom type, setting protonation state, fixing bond order, adding charge, and fixing side chain amide. Finally the prepared structure is minimized in order to remove the strain produced during the earlier protein preparation steps. The ‘protomol’ which is the defined binding pocket of receptor was generated using the co-crystallized ligand in the active site of ChAT. The prepared dataset compounds were docked into the active site of ChAT using Surflex-Dock GeomX (SFXC) approach and the compounds were ranked using Total_Score (−logK_d_). Similar strategy was used for docking analysis of novel designed compounds.

### HQSAR studies

Hologram QSAR (HQSAR) offers high quality QSAR models very rapidly and easily. In order to gain information about the individual contribution of molecular fragments of the potent molecule and to use the information along with the information obtained from molecular docking study to design novel potent analogs, HQSAR analysis was performed on the dataset compounds. HQSAR is based on the assumption that structure is a key determinant of all molecular property and the structural information encoded in the 2D molecular fingerprints can be thus successfully used for predict the activity of a molecule. As compared to traditional 2D fingerprints, molecular hologram contains additional information such as branched and cyclic fragments and stereochemistry of the molecule. Also, the molecular representation is purely binary in the case of hashed fingerprints while in the case of hologram, the bins contain information about the number of fragments hashed into each bin. The QSAR models were developed using various combinations of molecular fragments and fragment generation parameters for each hologram length in HQSAR module of SYBYL X 2.1.1 using partial least square (PLS) method^21^. In PLS method, the basic aim is to avoid over-fitted QSAR model by selecting the optimum number of components from PLS analysis with high cross validation correlation coefficient (q^2^) and low standard error of prediction. The generated models were cross validated using leave one out (LOO) and various statistical parameters such as optimum number of components (N) which provides highest q^2^ value, cross validation correlation coefficient (q^2^), coefficient of determination (r^2^). The various developed models on the basis of components for fragment generation are given in Table 3.

### Lead optimization and design of new analogues

The Muse^®^ Invent™ molecular design workflow was used to design several analogs based on the initial starting structures of active ligands from dataset. It contains a scoring function based on the pharmacophoric and shape based structural similarity^24^,^29^. The active compounds with pIC_50_ > 5 were used as not only reference molecules but also as seed structures. The other parameters were kept as default including 39 questionable structures and 99 undesirable structures to exclude. The top scored individuals from the population survives based on the cutoff score and further used to produce more generations until the optimization criteria are met. The scoring function, TriposScore based on ligands try to optimize the shape and pharmacophoric similarity to a set of lead structures. It also takes into account cLogP, MW, TPSA, Lipinski Counts, and Rotatable Bond Counts^30^.

### Purification of recombinant human ChAT

DYT media (16 g/l Tryptone, 10 g/l yeast extract, 5 g/l NaCl, 100 μg/ml ampicillin, 34 μg/ml chloramphenicol) was inoculated with a preculture of *E*. *Coli* BL21 Rosetta2 transformed with pProEXHTa-ChAT (a generous gift from Brian Shilton, Department of biochemistry, University of western Ontario, London, Canada). The bacteria were grown at 37 °C and 200 rpm until the optical density at 600 nm reached 0.5. After which 0.5 mM IPTG was added and His_6_-ChAT was expressed for circa 16 h at 18 °C. The bacteria was harvested and stored at −80 °C. His_6_-ChAT was purified with “Ni-NTA fast start Kit” (Qiagen) following the manufacturer’s instructions. The elution buffer was exchanged to storage buffer (10 mMTris pH 7.4, 500 mMNaCl, 10% (v/v) glycerol) using Amicon Ultra concentrators (Merck Millipore) with a molecular cutoff of 30 kDa. The protein preparation was aliquoted, frozen at dry ice and stored at −80 °C. The absence of contaminating proteins was determined using sodium dodecyl sulfate PAGE and Coomassie staining. The total protein concentration was measured with BioRad DC protein Assay (BioRad).

### Experimental enzymatic activity assays

A modified version of Ellman’s colorimetric assay was used for the enzymatic activity of BuChE and AChE, as described previously^31^,^32^. For BuChE activity, 50 μL/well of a 1:400 diluted solution of a pooled human plasma sample was used. For AChE activity, the 1:400 diluted pooled human plasma samples had been supplied with 50 ng/ml of purified AChE protein (Sigma). These were preincubated with 50 μL/wells of different concentrations of α-NETA for 30 minutes at room temperature. Then, 100 μL of a cocktail (Na/K phosphate buffer, containing 5,5′-dithiobis(2-nitrobenzoic acid) (DTNB, final concentration 0.4 mM) and butyrylthiocholine iodide (Sigma, final concentration 5 mM) or acetylthiocholine iodide (Sigma, final concentration 0.5 mM) were added and the changes in absorbance was monitored at one minutes intervals at 412 nm wavelength. For the AChE activity, the cocktail also contained the selective BuChE inhibitor ethopropazine (Sigma, final concentration 0.1 mM).

ChAT activity was measured with a new colorimetric assay as described previously^33^, using recombinant ChAT protein. Briefly, 20 μL/wells of 1.5 μg/ml of the recombinant ChAT was incubated with 20 μL/well of different concentrations of α-NETA for 30 minutes at room temperature in dilution buffer (10 mM Tris-HCl, pH 7.4, 150 mM NaCl, 1.0 mM EDTA, 0.05% (v/v) Triton X-100). Then 60 μl of a cocktail-A [dilution buffer containing choline chloride (Sigma, final concentration 250 μM), eserine (E8625, Sigma-Aldrich, final concentration 60 μM), acetyl coenzyme-A (A2181, Sigma-Aldrich, final concentration 50 μM), phosphotransacetylase (P2783, Sigma-Aldrich, final concentration 1.02 U/ml), lithium potassium acetyl-phosphate (#01409, Sigma-Aldrich, final concentration 12 mM)] was added to the samples. The final concentration of ChAT in the wells was 0.3 μg/mL.

In separate wells, 100 μl of a serial two-fold dilution of choline chloride (500–0 μM) was applied in triplicates, which were used as standards for determining choline concentration in the wells after reaction with ChAT. The plate was incubated for 20 minutes at 37 °C.

Then 50 μl of a cocktail-B [phosphate buffered saline, containing 0.93 U/ml choline oxidase (C5896, Sigma-Aldrich), 1/5000 U streptavidin-horseradish peroxidase, 6.3 mM phenol, and 3 mM 4-aminoantipyrine (A4382, Sigma-Aldrich)] was added to each sample including the standards. Absorbance was then monitored using a microplate spectrophotometer reader (Infinite M1000, Tecan) at 500 nm wavelength. ChAT activity (nmol/min/mg of recombinant protein) was calculated according to the following formula: ChAT activity [Ch_BL_-Ch_S_]/*t*/*m*, where Ch_BL_ is the measured number of mole of choline in control wells lacking the inhibitor and ChAT, Ch_S_ is the measured number of mole of choline in the sample wells, the *t* is the incubation time and *m* is the mass of ChAT protein added per sample well. Inhibition is given as compared to a ChAT sample incubated with only buffer.

## Additional Information

**How to cite this article**: Kumar, R. *et al*. Novel ligands of Choline Acetyltransferase designed by in silico molecular docking, hologram QSAR and lead optimization. *Sci. Rep*. **6**, 31247; doi: 10.1038/srep31247 (2016).

## Figures and Tables

**Table 1:**
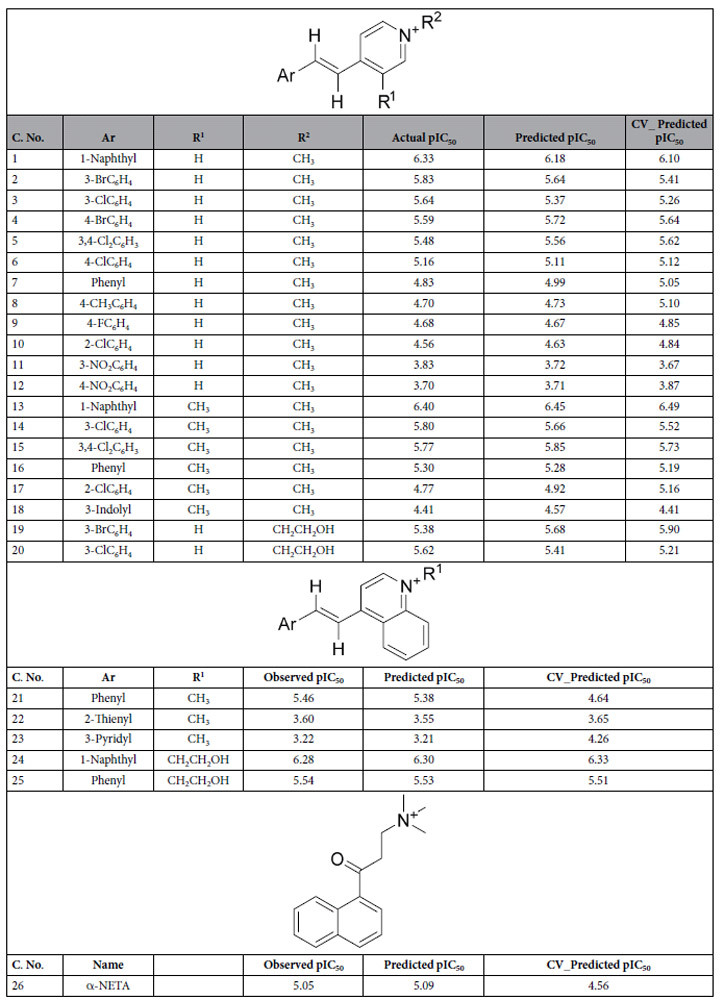
Dataset used in the HQSAR and molecular docking study.

**Table 2:** Hologram QSAR analysis of dataset compounds using different fragment components.

S. No.	Components	Best Length	Number of Components	R^2^ (Std.Error)	Q^2^ (Std. Error CV)	Ensemble Q^2^ Ensemble Std.Error (CV)	No. of Models in Ensemble	No. of Models Dropped from Ensemble
01	A/B	151	5	0.90 (0.30)	0.68 (0.54)	0.63 (0.58)	6	0
02	A/B/C	151	6	0.98 (0.14)	0.80 (0.43)	0.72 (0.50)	6	0
03	A/B/C/H	257	6	0.91 (0.30)	0.58 (0.64)	0.42 (0.73)	6	0
04	A/B/C/Ch	151	6	0.98 (0.15)	0.81 (0.43)	0.71 (0.51)	6	0
05	A/B/C/H/Ch	353	6	0.91 (0.29)	0.59 (0.63)	0.42 (0.73)	6	0
06	A/C/DA	307	5	0.91 (0.29)	0.65 (0.56)	0.53 (0.64)	6	0
07	A/B/C/H/DA	257	6	0.89 (0.32)	0.51 (0.69)	0.34 (0.79)	6	0
08	A/B/H/	199	6	0.91 (0.29)	0.62 (0.60)	0.51 (0.66)	6	0
09	A/B/H/DA	307	6	0.92 (0.27)	0.51 (0.69)	0.38 (0.74)	5	1
10	A/B/C/DA	97	5	0.92 (0.27)	0.73 (0.49)	0.67 (0.54)	6	0
11	A/B/Ch/DA	353	4	0.85 (0.37)	0.58 (0.60)	0.38 (0.72)	6	0
12	A/B/H/Ch	307	6	0.94 (0.24)	0.67 (0.56)	0.57 (0.62)	6	0
13	A/B/DA	257	4	0.85 (0.37)	0.53 (0.64)	0.40 (0.71)	6	0
14	A/B/Ch	353	5	0.91 (0.29)	0.72 (0.51)	0.63 (0.58)	6	0

q2, cross-validated correlation coefficient; r2, non-cross-validated coefficient of determination; SEE, standard error of estimate; n, number of statistical components; HL, hologram length; A, atoms; B, bonds; C, connection; H, hydrogen atoms; Ch, chirality; DA, donor and acceptor.

**Table 3:** Results of CScore analysis of ChAT inhibitors in the dataset.

C. No.	pIC_50_	Total_Score −log(Kd)	Crash	Polar
1	6.33	6.07	−2.57	0
2	5.83	4.37	−1.33	0
3	5.64	4.59	−1.21	1.10
4	5.59	4.50	−1.10	0
5	5.48	4.07	−0.45	0.73
6	5.16	4.38	−0.93	0.09
7	4.83	4.90	−1.66	0
8	4.7	4.85	−0.40	0
9	4.68	4.36	−0.82	0
10	4.56	4.82	−1.01	0.00
11	3.83	4.80	−0.90	0.85
12	3.7	4.86	−1.08	0.89
13	6.4	6.09	−1.01	0
14	5.8	4.70	−1.68	0.81
15	5.77	5.18	−0.52	0.00
16	5.3	4.80	−0.73	0
17	4.77	5.56	−1.33	0
18	4.41	6.46	−1.54	0.01
19	5.38	5.57	−2.09	0.84
20	5.62	6.27	−1.68	1.03
21	5.46	5.94	−2.05	0
22	3.6	5.47	−1.02	0.01
23	3.22	6.57	−1.49	0
24	6.28	8.12	−2.28	1.82
25	5.54	5.77	−3.48	0
α-NETA	5.05	5.30	−3.45	0.08

**Table 4:** Novel designed compounds along with their docking score against ChAT.

Name	Total_Score	Polar	Crash	MaxTanRef	MolWt	SurflexSim	HBA Count	HBD Count	RotBond Count	TPSA	ALogP	Score
G030_C020	8.27	0	−2.05	0.33	357.60	0.59	1	0	13	38.19	6.85	4.28
G036_C015	7.37	0.13	−2.22	0.44	311.50	0.60	1	0	12	12.89	7.04	3.64
G028_C030	6.98	0	−2.73	0.35	277.47	0.62	1	0	10	38.19	4.95	4.63
G033_C006	6.96	0	−2.48	0.37	311.50	0.61	1	0	12	12.89	7.04	3.74
G007_C053	7.04	1.4	−4.31	0.28	304.39	0.55	3	0	4	32.67	4.37	4.62
G013_C037	8.68	2.16	−2.29	0.23	304.34	0.59	6	1	6	69.15	1.26	4.72
G014_C032	8.05	1.51	−2.19	0.24	304.34	0.59	6	1	6	69.15	1.00	4.89
G016_C028	7.08	1.45	−0.95	0.27	318.37	0.59	6	1	7	69.15	1.25	5.37
G031_C030	7.83	0.00	−3.73	0.34	357.60	0.59	1	0	13	38.19	6.71	4.25
G037_C047	7.42	0.55	−2.04	0.23	328.43	0.61	3	0	3	52.99	3.82	4.50

## References

[b1] WHO 10 facts on dementia < http://www.who.int/features/factfiles/dementia/en/> (2015).

[b2] Mesulam M. M. Cholinergic Circuitry of the Human Nucleus Basalis and Its Fate in Alzheimer’s Disease. J. Comp. Neuro. 521, 4124–4144 (2013). 10.1002/cne.23415 PMC4175400 23852922

[b3] Bohnen N. I. . Cortical cholinergic function is more severely affected in parkinsonian dementia than in Alzheimer disease: an *in vivo* positron emission tomographic study. Arch. Neurol. 60, 1745–1748 (2003). 14676050 10.1001/archneur.60.12.1745

[b4] Yates C. M., Simpson J., Maloney A. F., Gordon A. & Reid A. H. Alzheimer-like cholinergic deficiency in Down syndrome. Lancet 2, 979 (1980). 6107618 10.1016/s0140-6736(80)92137-6

[b5] Mesulam M. M. The cholinergic innervation of the human cerebral cortex. Prog. Brain Res. 145, 67–78 (2004). 14650907 10.1016/S0079-6123(03)45004-8

[b6] Wilcock G. K., Esiri M. M., Bowen D. M. & Smith C. C. Alzheimer’s disease. Correlation of cortical choline acetyltransferase activity with the severity of dementia and histological abnormalities. J. Neurol. Sci. 57, 407–417 (1982). 7161627 10.1016/0022-510x(82)90045-4

[b7] Kim A. R., Rylett R. J. & Shilton B. H. Substrate binding and catalytic mechanism of human choline acetyltransferase. Biochemistry 45, 14621–14631 (2006). 17144655 10.1021/bi061536l

[b8] Esiri M. M., Pearson R. C., Steele J. E., Bowen D. M. & Powell T. P. A quantitative study of the neurofibrillary tangles and the choline acetyltransferase activity in the cerebral cortex and the amygdala in Alzheimer’s disease. J. Neurol. Neurosurg. Psychiatry 53, 161–165 (1990). 2313304 10.1136/jnnp.53.2.161 PMC487958

[b9] Nordberg A., Rinne J. O., Kadir A. & Langstrom B. The use of PET in Alzheimer disease. Nat. Rev. Neurol. 6, 78–87 (2010). 20139997 10.1038/nrneurol.2009.217

[b10] Tracey K. J. Reflex control of immunity. Nat. Rev. Immunol. 9, 418–428 (2009). 19461672 10.1038/nri2566 PMC4535331

[b11] Cheng K. . Acetylcholine release by human colon cancer cells mediates autocrine stimulation of cell proliferation. Am. J. Physiol. Gastrointest. Liver. Physiol. 295, G591–G597 (2008). 18653726 10.1152/ajpgi.00055.2008 PMC2536781

[b12] Nachmansohn D. & Machado A. L. The formation of acetylcholine. A new enzyme: “Choline Acetylase”. J. Neurophysiol. September 1, 397–403 (1943).

[b13] Sastry B. V., Jaiswal N., Owens L. K., Janson V. E. & Moore R. D. 2-(alpha-Naphthoyl)ethyltrimethylammonium iodide and its beta-isomer: new selective, stable and fluorescent inhibitors of choline acetyltransferase. J. Pharmacol. Exp. Ther. 245, 72–80 (1988). 3361452

[b14] Mehta N., Musso D. & White H. Water soluble choline acetyltransferase inhibitors: SAR studies. Eur. J. Med. Chem. 20, 443–446 (1985).

[b15] Bajorath J. Integration of virtual and high-throughput screening. Nat. Rev. Drug. Discov. 1, 882–894 (2002). 12415248 10.1038/nrd941

[b16] Green K. D., Porter V. R., Zhang Y. & Garneau-Tsodikova S. Redesign of cosubstrate specificity and identification of important residues for substrate binding to hChAT. Biochemistry 49, 6219–6227 (2010). 20560540 10.1021/bi1007996

[b17] Kumar R. & Kumar M. 3D-QSAR CoMFA and CoMSIA studies for design of potent human steroid 5α-reductase inhibitors. Med. Chem. Res. 22, 105–114 (2013).

[b18] Malla P., Kumar R. & Kumar M. Validation of formylchromane derivatives as protein tyrosine phosphatase 1B inhibitors by pharmacophore modeling, atom-based 3D-QSAR and docking studies. Chem. Biol. Drug. Des. 82, 71–80 (2013). 23506477 10.1111/cbdd.12135

[b19] Zhang C., Du C., Feng Z., Zhu J. & Li Y. Hologram quantitative structure activity relationship, docking, and molecular dynamics studies of inhibitors for CXCR4. Chem. Biol. Drug. Des. 85, 119–136 (2015). 24923360 10.1111/cbdd.12377

[b20] Primi M. C. . Convergent QSAR studies on a series of NK receptor antagonists for schizophrenia treatment. J. Enzyme Inhib. Med. Chem. 1–12 (2015). 10.3109/14756366.2015.1021250 25856571

[b21] Kumar R., Kumar A., Jain S. & Kaushik D. Synthesis, antibacterial evaluation and QSAR studies of 7-[4-(5-aryl-1, 3, 4-oxadiazole-2-yl) piperazinyl] quinolone derivatives. Eur. J. Med. Chem. 46, 3543–3550 (2011). 21689870 10.1016/j.ejmech.2011.04.035

[b22] Radosevic-Stasic B. . Immunological consequences of lesions of nucleus basalis in rats. Int. J. Neurosci. 51, 325–327 (1990). 2279895 10.3109/00207459008999733

[b23] Gao J., Wu X. H., Dong W. L. & Wang S. Q. A series of propofol analogs design by targeting pentameric ligand-gated ion channel in silico method. Protein Pept. Lett. 20, 1238–1245 (2013). 23848595 10.2174/09298665113209990044

[b24] Damewood J. R. Jr., Lerman C. L. & Masek B. B. NovoFLAP: A ligand-based de novo design approach for the generation of medicinally relevant ideas. J. Chem. Inf. Model. 50, 1296–1303 (2010). 20586434 10.1021/ci100080r

[b25] Chandrasekaran V., McGaughey G. B., Cavallito C. J. & Bowen J. P. Three-dimensional quantitative structure-activity relationship (3D-QSAR) analyses of choline acetyltransferase inhibitors. J. Mol. Graph. Model. 23, 69–76 (2004). 15331055 10.1016/j.jmgm.2004.04.002

[b26] Everett J. R. Academic drug discovery: current status and prospects. Expert Opin. Drug Discov. 10, 937–944 (2015). 26088126 10.1517/17460441.2015.1059816

[b27] Jain A. N. Surflex: fully automatic flexible molecular docking using a molecular similarity-based search engine. J. Med. Chem. 46, 499–511 (2003). 12570372 10.1021/jm020406h

[b28] Warren G. L., Do T. D., Kelley B. P., Nicholls A. & Warren S. D. Essential considerations for using protein–ligand structures in drug discovery. Drug Discov. Today 17, 1270–1281 (2012). 22728777 10.1016/j.drudis.2012.06.011

[b29] Liu Q., Masek B., Smith K. & Smith J. Tagged fragment method for evolutionary structure-based de novo lead generation and optimization. J. Med. Chem. 50, 5392–5402 (2007). 17918924 10.1021/jm070750k

[b30] Bös F., Uhrig U., Masek B. B. & Damewood J. R. Muse + TriposScore: a ligand-based de novo design approach. j. Cheminform. 3, P26–P26 (2011).

[b31] Darreh-Shori T., Brimijoin S., Kadir A., Almkvist O. & Nordberg A. Differential CSF butyrylcholinesterase levels in Alzheimer’s disease patients with the ApoE epsilon4 allele, in relation to cognitive function and cerebral glucose metabolism. Neurobiol. Dis. 24, 326–333, 2006). 16973370 10.1016/j.nbd.2006.07.013

[b32] Darreh-Shori T. . Inhibition of acetylcholinesterase in CSF versus brain assessed by 11C-PMP PET in AD patients treated with galantamine. Neurobiol. Aging 29, 168–184 (2008). 17196712 10.1016/j.neurobiolaging.2006.09.020

[b33] Vijayaraghavan S. . Regulated Extracellular Choline Acetyltransferase Activity- The Plausible Missing Link of the Distant Action of Acetylcholine in the Cholinergic Anti-Inflammatory Pathway. PLoS One 8, e65936 (2013). 23840379 10.1371/journal.pone.0065936 PMC3686815

